# Plate osteosynthesis versus non-surgical treatment in displaced proximal humerus fractures—long term functional outcome and quality of life

**DOI:** 10.1007/s00590-025-04290-9

**Published:** 2025-04-29

**Authors:** Lisa Klute, Christian Pfeifer, Arne Berner, Volker Alt, Maximilian Kerschbaum, Leopold Henssler

**Affiliations:** 1 University Hospital Regensburg, Regensburg, Germany; 2 Clinic of Trauma and Hand Surgery, Altötting, Germany; 3 Clinic of Trauma Surgery, Bad Neustadt, Germany

**Keywords:** Proximal humerus fracture, Shoulder, PHILOS plate, Fractures of the elderly

## Abstract

**Purpose:**

This study aimed to assess the long-term outcomes of patients treated with plate osteosynthesis versus non-operative treatment for proximal humerus fractures (PHF) after a mean follow-up period of 10 years.

**Methods:**

A retrospective cohort study was conducted, including patients with PHF treated between 2004 and 2014. Patients were divided into two groups: those who underwent plate osteosynthesis (PO) and those managed non-surgically (NO). Functional outcomes, including range of motion, strength, and patient-reported quality of life were evaluated using standardized assessments such as the Constant-Murley score and Short-Form-36 (SF-36) questionnaire.

**Results:**

A total of 241 patients (161 in the Surgical Group and 80 in the Non-Operative Group) were included in the study. With a mean follow-up of 10.4 ± 3.1 years, both groups demonstrated comparable functional outcomes. The Constant-Murley score in the Surgical Group was 53.5 ± 21.8 compared to 60.1 ± 24.2 in the Non-Operative Group (*p* = 0.225). Complication rates were significantly higher in the PO group. The revision rate for patients treated with plate osteosynthesis was 37.9%. Patient-reported Quality of Life, assessed using the SF-12 questionnaire, revealed no significant differences between the Surgical and Non-Operative Groups.

**Conclusion:**

This long-term follow-up study demonstrates that after a minimum of 5 years, there were no significant differences in functional outcomes or quality of life between patients treated with plate osteosynthesis and those who were managed non-operatively for displaced proximal humerus fractures. Both treatment approaches can offer favorable results, and the choice of treatment should consider individual patient characteristics and preferences.

## Background

Proximal humerus fractures (PHF) represent a common and hitherto challenging traumatic condition encountered in clinical practice [1, 2]. These fractures can result from various high-impact accidents, including falls, sports-related injuries, or as fragility fractures in the elderly patient [3]. With the global increase in the aging demographic, the incidence of PHF is expected to rise [4, 5], making its management a vital concern. Managing PHF has been a subject of considerable research and clinical debate [6], with a wide spectrum of treatment options, ranging from non-surgical approaches to various surgical interventions. Among the surgical strategies, the use of the plate osteosynthesis has gained relevance as an effective tool for stabilizing and repairing displaced fractures of the proximal humerus [7].

Despite the decades of improvement in surgical expertise and scientific inquiry, the optimal management approach for PHF remains a contentious issue [6]. The choice between surgical intervention and non-surgical treatment is often influenced by factors such as fracture complexity, patient demographics, and surgeon preference [8–10]. Consequently, the need for evidence-based decision-making in this domain persists [2].

Treatment options for PHF range from non-surgical methods to surgical interventions like closed reduction and percutaneous pinning, open reduction and internal fixation using various implants (for example plate osteosynthesis or intramedullary nailing), and reverse or anatomic shoulder arthroplasty. While non-surgical treatment for displaced PHF has gained popularity in the last years [11], the use of as well as the scientific interest on reverse shoulder arthroplasty in fracture situations also increased recently [12]. However, head-preserving interventions, including osteosynthesis or non-surgical treatment, remain crucial therapeutic modalities across all age groups, particularly in situations where fractures are reconstructable [2]. Although numerous investigations focusing on short- to mid-term outcomes for either non-surgical or surgical approaches [13], studies delving into long-term results are scarce.

This study aims to address the critical knowledge gap on long-term functional outcomes and quality of life by evaluating two important head-preserving treatments of PHF: plate osteosynthesis and non-surgical management.

## Methods

### Inclusion and exclusion criteria

An analysis was conducted on all patients treated for proximal humerus fractures at a level-I trauma center between 2004 and 2014. The primary dataset for analysis consisted of 522 fractures, including 2-part, 3-part, and 4-part fractures. Exclusion criteria encompassed patients subjected to non-head-preserving treatments (for example primary shoulder arthroplasty), those below 18 or older than 99 years of age, and individuals with pathologic fractures. Additionally, only type 4 and type 5 fractures were considered. Subsequently, patients undergoing primary treatment with intramedullary nailing were also excluded.

**Fig. 1:**
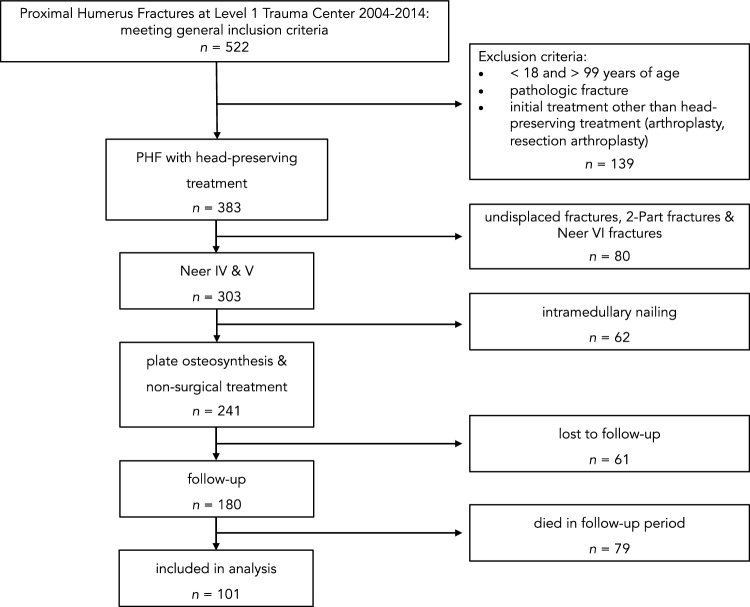
Flowchart of case inclusion and exclusion. The study population only consisted of patients with PHF and a follow-up of at least 5 years

Following a minimum follow-up period of 5 years, attempts were made to contact the remaining 241 patients for the evaluation of functional outcomes and quality of life. Complete functional and clinical follow-up data were successfully obtained from 101 patients, while 61 were lost to follow-up and 79 had deceased (Fig. 1). The study protocol and the procedures have been approved by the Ethics Committee of the University (20-1733-101).

### Surgical treatment by plate osteosynthesis (PO)

Treatment decisions were guided by factors including patient age, comorbidities, associated orthopedic injuries necessitating surgery, and specific fracture characteristics, such as medial hinge abduction, comminution, or varus impaction. Patients with a higher demand for independence were more likely to undergo surgical intervention. Operative treatment was performed with the patient in the beach chair position by senior surgeons. Following sterile preparation and draping, a deltopectoral approach was used to access the fracture site. After achieving fracture reduction, confirmed by intraoperative fluoroscopy, plate osteosynthesis (Proximal Humeral Internal Locking System, DePuy Synthes®, Massachusetts, USA) was performed according to the manufacturer’s guidelines. Postoperatively, the shoulder was immobilized using a Gilchrist sling for 14 days, with a gradual reduction in wearing time during this period. Functional therapy commenced with pendulum exercises on the first or second postoperative day, with active-assistive shoulder motion exercises introduced progressively over the following days.

### Non-surgical treatment (NO)

Patients who received non-operative treatment had their affected shoulder immobilized using a Gilchrist sling for 14 days. Functional therapy began on the fifth day with pendulum exercises. Starting after 14 days, the rehabilitation program was intensified to include passive and active-assistive shoulder motion exercises, guided by a physiotherapist and supplemented by self-exercises aimed at gradually increasing the range of motion. During the first six weeks, follow-up X-rays were scheduled during the first and second week, and after 3 and 6 weeks to monitor fracture healing. Weight bearing of the arm was strictly avoided for the initial 6 weeks, after which gradual loading was introduced. Pain management was continuously adjusted to the patient’s needs throughout the rehabilitation period.

### Parameter investigation

For this study, a comprehensive set of relevant information was collected from the patient cohort. The gathered data included various factors such as age, sex, date of accident, concomitant injuries, pre-existing comorbidities, and treatment details. Specifically, the type of treatment administered was recorded, distinguishing between conservative management and surgical interventions. Surgeries were performed with open reduction and internal fixation using the PHILOS Plate (Proximal Humeral Internal Locking System, DePuy Synthes®).

To assess the presence and impact of comorbidities, the Charlson Comorbidity Index (CCI) was employed as a standardized measure. This facilitated the evaluation of the overall burden of chronic conditions in the patient population. Concerning fracture-related data, X-rays of the affected shoulder in true anteroposterior and Y-view orientations, and in most cases, CT scans were utilized. These radiographs underwent a thorough radiological evaluation using the widely accepted Neer classification system [14]. This classification system allowed the categorization of fractures based on specific characteristics, enabling a standardized approach to analyzing and interpreting the data. The fractures were classified by two senior residents, with agreement on classification results. The classification was based on the assessment of the displacement of the four key fragments: the humeral head, the lesser tubercle, the greater tubercle, and the shaft. According to Neer’s classification [8], only displaced proximal humerus fractures with a displacement of 1 cm or an angulation of 45° in key fragments were included for further analysis. Specifically, fractures with displacement of the lesser or greater tubercles (Neer type IV and V) were considered, while non-displaced fractures (Neer type I) were primarily excluded. This focus on Neer type IV and V fractures is particularly relevant as they are often central to the ongoing debate between non-surgical treatment and surgical treatment with plate osteosynthesis. While there is consensus that Neer I fractures can be managed non-surgically and Neer VI fractures should be treated surgically, Neer II (anatomical neck) and III (surgical neck) fractures encompass a heterogeneous group with a wide range of potential treatment options. Following a minimum follow-up period of 5 years, members of the patient cohort were proactively contacted through a combination of telephone and written correspondence. Shoulder function was evaluated using both the Constant Score (CMS) and the age-adapted Constant Score (CMSa), incorporating criteria such as pain (15 points), range of motion (40 points), muscle strength (25 points), and activity of daily living (20 points).

The limitations of the affected arm in daily activities were appraised through the QuickDASH questionnaire, which encompasses 11 questions on abilities in daily activities and sports. Additionally, quality of life was assessed using the Short-Form 12 questionnaire (SF-12), with a focus on its subscales for physical health (SF-12-PCS) and mental health (SF-12-MCS).

### Statistical analysis

Statistical analysis was carried out using SPSS software package version 25 (SPSS Inc., Chicago, IL, USA). The chi-square independence test was performed to compare categorical variables. The independent t-test was used to compare continuous variables after determining that all variables are normally distributed (Kolmogorov–Smirnov normality test). *p*-values < 0.05 were considered significant. All graphs are displayed with mean values and 95% confidence intervals.

## Results

A total of 241 patients were included in the study, with 161 in the surgical Group (plate osteosynthesis) and 80 in the non-surgical group (managed non-surgically). The mean follow-up duration was 10.4 ± 3.1 years. While 79 patients had passed away, quality of life assessment as well as functional scores were obtained for 101 patients (79 PO and 22 NO). In comparison patients were significantly older and had more comorbidities in the deceased group (Table 1).

**Table 1:** Epidemiology of the study collective, patients lost to follow-up and deceased patients

*Chi^2^-test ** independent samples *t*-test		Total (*n* = 241)	Dead (*n* = 79)	Lost to follow-up (*n* = 61)	Responder (*n* = 101)	*P* value
Age [years]		72.8 ± 16.6	84.4 ± 11.2	68.8 ± 16.1	66.1 ± 15.6	< 0.05**
Sex [*n*]		♂ = 89 ♀ = 152	♂ = 24 ♀ = 55	♂ = 26 ♀ = 35	♂ = 39 ♀ = 62	0.297*
Fracture classification [*n*]	*Neer IV*	123	39	26	58	0.177*
	*Neer V*	118	40	35	43	
Charlson comorbidity index [mean]		0.8 ± 0.7	1.6 ± 1.7	0.4 ± 0.7	0.4 ± 1.0	< 0.05**
Therapy [*n*]	*Plate osteosynthesis*	161	44	38	79	0.388*
	*Non-surgical*	80	35	23	22	

Values that are in bold indicate indicate statistically significant differences (*p* < 0.05)

Functional outcomes were assessed using the Constant-Murley score (CMS) and its age and sex-adapted version (CMSa), as well as the QuickDASH (QD) questionnaire. Quality of life assessments included the Short-Form 12 questionnaire.

### Neer IV & V fractures

**Fig. 2:**
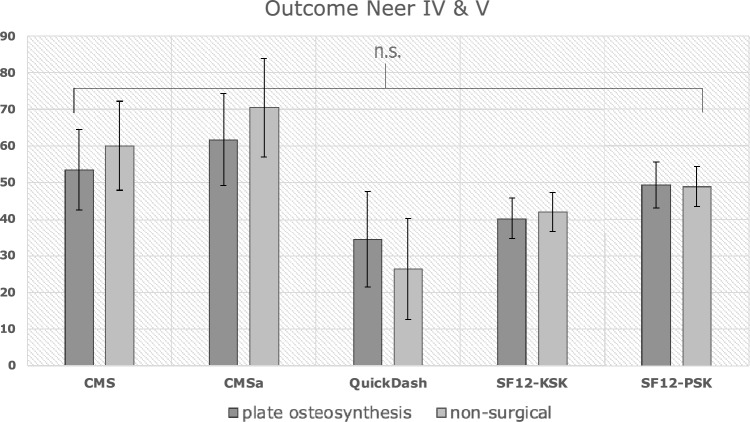
Functional Outcome and Quality of Life in Neer IV and V fractures

The results revealed comparable long-term functional outcomes between the surgical and non-surgical groups (Fig. 2). The Constant-Murley score in the Surgical Group was 53.5 ± 21.8, while in the non-surgical group, it was 60.1 ± 24.2 (*p* = 0.225). Similarly, the age and sex-adapted Constant-Murley score (CMSa) showed no significant difference between the groups, with scores of 61.7 ± 25.2 in the surgical group and 70.5 ± 27.1 in the non-surgical group (*p* = 0.157).

Assessment of the QuickDASH (QD) questionnaire demonstrated comparable limitations in daily activities between the surgical and non-surgical groups, with scores of 34.4 ± 26.2 and 26.4 ± 27.6, respectively (*p* = 0.213).

Patient-reported Quality of Life, assessed using the SF-12 questionnaire, revealed no significant differences in physical and mental health components between the surgical 40.6 ± 10.1 (PCS), 49.3 ± 11.9 (MCS) and non-surgical 42.1 ± 10.6 (PCS), 48.9 ± 12.4 (MCS) groups, demonstrating p-values of 0.486 (PCS) and 0.854 (MCS).

### Neer IV fractures

**Fig. 3:**
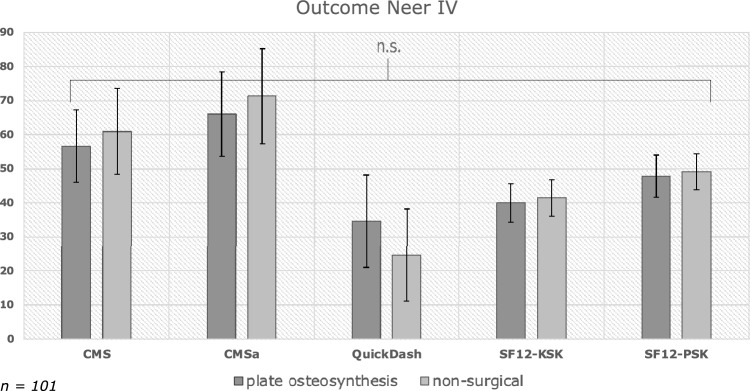
Analysis of functional outcome and life quality in Neer IV fractures

In the context of Neer IV fractures, the investigation yielded comparable long-term functional outcomes within the PO and NO Groups (refer to Fig. 3). The Constant-Murley score for the surgical Group was 56.7 ± 21.2, contrasting with 61.0 ± 25.1 in the non-surgical group (*p* = 0.500). Additionally, the adapted Constant-Murley score displayed no statistically significant variance between the groups, recording scores of 66.0 ± 24.6 in the surgical group and 71.3 ± 28.0 in the non-surgical Group (*p* = 0.466).

Evaluation of the QuickDASH questionnaire, indicated similar outcomes between the surgical and non-surgical groups, registering scores of 34.7 ± 27.1 (PO) and 24.6 ± 27.2 (NO) (*p* = 0.191).

Patient-reported Quality of Life, as gauged by the SF-12 questionnaire, disclosed no noteworthy differences in physical and mental health components between the surgical 40.0 ± 10.2 (PCS), 48.0 ± 12.3 (MCS) and non-surgical 41.5 ± 10.2 (PCS), 49.1 ± 10.7 (MCS) groups, demonstrating p-values of 0.630 (PCS) and 0.711 (MCS).

### Neer V fractures

**Fig. 4:**
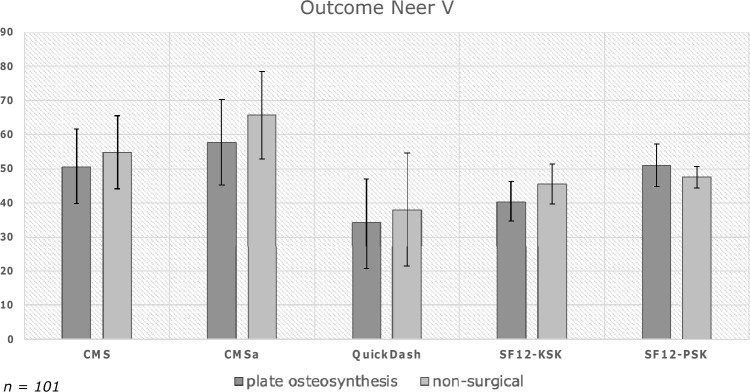
Subgroup analysis of Neer V fractures

For Neer V fractures, a subgroup analysis was performed and showed comparable long-term functional outcomes between patients with Neer V fractures treated with Plate Osteosynthesis and those managed non-surgically (Fig. 4). The Constant-Murley score for the surgical group averaged 50.5 ± 22.2, while the non-surgical Group exhibited a mean of 54.8 ± 21.3 (*p* = 0.668). Similarly, the adjusted Constant-Murley score showed no statistically significant differences between the groups, with scores of 57.6 ± 25.3 in the surgical group and 65.7 ± 25.1 in the non-surgical group (*p* = 0.771).

Analysis of the QuickDASH questionnaire highlighted similar outcomes between the surgical and non-surgical groups, revealing scores of 34.2 ± 25.5 (PO) and 38.0 ± 33.2 (NO) (*p* = 0.629).

Assessment of Patient-reported Quality of Life, conducted through the SF-12 questionnaire, revealed no discernible disparities in physical and mental health components between the surgical (PCS: 40.2 ± 12.3, MCS: 51.0 ± 12.5) and non-surgical (PCS: 45.5 ± 11.7, MCS: 47.5 ± 6.3) groups, yielding p-values of 0.638 (PCS) and 0.113 (MCS).

### Complication & revisions

Complication rates were notably higher in the surgical Group. Capsulitis (27.7%) and restricted motion (9.9%) were the most frequently occurring complications (Table 2). The revision rate for plate osteosynthesis patients was 37.9%, including material removal in 32.9% and other surgical interventions in 5% of cases (Table 3). In contrast, the non-surgical group had a conversion rate to surgery of 0% over the entire study period (*p* < 0.05).

**Table 2:** Complications of the analyzed patients

Complications in neer IV & V fractures	*n* [%]
Total	46 [45.5]
Capsulitis	28 [27.7]
Restricted motion	10 [9.9]
Secondary dislocation	2 [2.0]
Implant failure	2 [2.0]
Humeral head necrosis	1 [1.0]
Non-union	1 [1.0]
Nerval irritation	1 [1.0]
Infection	1 [1.0]

**Table 3:** Revision surgeries in surgically treated patients

Revision surgery in philos plate osteosynthesis		*n* [%]
Total		30 [37.9]
Implant removal		26 [32.9]
	Additional arthroscopic arthrolysis	13
	Additional open arthrolysis	11
Shoulder arthroplasty		1 [1.3]
Revision plate osteosynthesis		2 [2.6]
Resection arthroplasty		1 [1.3]

## Discussion

The key findings of our study are:

- No statistically relevant differences in functional outcome and quality of life in treatment with plate osteosynthesis and non-surgical treatment for displaced proximal humerus fractures after a mean follow-up period of 10 years
- Significantly more complications and revisions in patients treated with plate osteosynthesis compared to non-surgical treatment in Neer IV & V fractures

The findings of this conducted long-term follow-up study align with the ProFHER trial, which investigated the outcomes of surgical versus non-surgical treatment for PHF at 2 and 5 years [15, 16]. Both studies underscore the complexity of decision-making in PHF management. The ProFHER trial, also focusing on displaced PHF, reported no significant differences in functional outcomes between surgical and non-surgical groups at the 2 and 5 year mark [15, 16]. Our study extends this perspective by assessing patients over a mean follow-up period of 10 years, revealing that the functional outcomes remain comparable between plate osteosynthesis and non-surgical groups. These consistent results over an extended duration emphasize the reliability and stability of outcomes, with our study as a retrospective protocol supporting the shorter follow-up times of the randomized controlled ProFHER trial [16].

Numerous publications have delved into the comparative effectiveness of different surgical or non-surgical techniques for PHF, particularly increasing its focus on Philos plating in the last years [2, 11]. While Philos plating has gained popularity for its biomechanical advantages in stabilizing fractures, there have been a lot of publications pointing to the high complication rates and need for revision surgery [17, 18]. Relevant research explored the nuanced factors influencing treatment outcomes, such as fracture pattern, patient age, and surgeon expertise, to guide clinicians in tailoring interventions based on individual patient characteristics [2, 10].

Mortality outcomes following plate osteosynthesis for PHF have been an emerging area of interest [19], for PHF still count for one of the most prevalent fractures in the elderly [20]. The significantly higher complication rates in the plate osteosynthesis group, as demonstrated in our findings, warrant further investigation into the long-term consequences of these complications, including potential associations with mortality [21]. Integrating mortality data into future evaluation could enhance our understanding of the comprehensive implications of different treatment modalities on patients with PHF [19].

Despite the observed complications associated with plate osteosynthesis, the relevance of this treatment modality definitely persists in the context of head-preserving PHF management [2]. The high revision rate in the PO group underscores the need for meticulous patient selection and surgical technique [2, 19]. There have been relevant studies supporting the superiority of plate osteosynthesis in PHF with complex fracture entities [22–24]. The decision to pursue surgical intervention should weigh the potential for complications against the demonstrated comparable functional outcomes with non-surgical management [2, 15].

Hitherto, the significance of plate osteosynthesis is unquestionable in proximal humerus fractures associated with a heightened risk of avascular necrosis, including head-split fractures, dislocation fractures, and open fractures, as well as concomitant neurovascular injuries. This holds particular importance in the treatment of young patients, where preserving the humeral head is particularly relevant [11, 25]. Despite the 33% hardware removal rate in our study collective, primarily due to restricted range of motion, post-traumatic shoulder stiffness, and soft tissue irritation caused by the implant, plate osteosynthesis (PO) remains a crucial surgical option for specific indications. Its effectiveness in providing stable fracture fixation justifies its continued use in appropriately selected cases [11, 25].

Our study emphasizes the importance of individualized treatment algorithms for PHF. The choice between plate osteosynthesis and non-surgical management should be guided by a thorough assessment of patient-specific factors, including age, comorbidities, fracture characteristics, and patient preferences [2, 10]. Tailoring treatment strategies based on individual patient profiles aligns with the concept of personalized medicine, promoting the best possible outcomes while minimizing unnecessary interventions and associated risks as shown by Spross et al. [26]. Future research should support the development and validation of decision-making algorithms that integrate diverse patient parameters, facilitating a more nuanced and patient-centered approach to PHF management [2, 27].

### Strengths and limitations

This investigation of long-term outcomes of proximal humerus fractures presents several notable strengths that contribute to the robustness of our findings. Firstly, the number of patients: We initially included 241 patients, and despite the challenges of a long follow-up period, with 61 patients lost to follow-up and 79 deaths, we successfully obtained comprehensive outcome data with complete functional outcome and quality of life parameters for 101 patients over a prolonged mean follow-up period of 10.4 ± 3.1 years.

This extended duration facilitated the capture of nuanced trends in functional recovery and complication rates, providing a comprehensive understanding of the outcomes associated with plate osteosynthesis and non-surgical management. The utilization of standardized assessments, particularly the CMS and the SF-12 questionnaire, ensured objective and comparable measurements of shoulder function and patient-reported quality of life. These widely accepted metrics enhance the validity of our results and allow for meaningful comparisons with existing literature.

While our study offers valuable insights, it is essential to acknowledge its inherent limitations. The study's limitations include a significant lost to follow-up rate, which may impact the generalizability of the findings. The retrospective nature of the design introduces the possibility of selection bias and the reliance on existing medical records. The availability and completeness of historical data may vary, influencing the accuracy of our assessments. There is definitely a possibility of selection bias, where patients with more severe health conditions may have been more likely to receive non-surgical treatment, while those requiring quicker physical recovery may have been directed towards surgical intervention. The indications for choosing either surgical or non-operative treatment were not standardized across the study population due to the retrospective design. While it is well-recognized that surgeons’ preferences can significantly influence treatment decisions in clinical practice, at our trauma center, these decisions were primarily guided by evidence-based factors, including the patient’s age, comorbidities, pain severity, and specific fracture characteristics, such as medial hinge abduction, comminution, or varus impaction. Additionally, the absence of randomization may introduce confounding variables that could impact the internal validity of our findings. An additional important limitation is the lack of an assessment of reduction quality, which is essential for a comprehensive understanding of the lower Constant scores observed in the plate osteosynthesis group. The primary objective of this study was to investigate long-term outcomes in patients with PHF treated either surgically with plate osteosynthesis or non-surgically, rather than to focus on radiological findings or the influence of surgical precision in postoperative imaging. While certain studies emphasize that accurate reduction of the 4 relevant parts of the proximal humerus is relevant for optimizing both functional outcomes and patient-reported satisfaction [28], other research suggests that the presence or absence of displacement following surgical treatment of PHF does not significantly impact the average side-adapted Constant score [29]. Additionally, considering our mean follow-up duration of 10 years, it is anticipated that any complications related to suboptimal reduction, or the onset of avascular necrosis (AVN) would have manifested as limited range of motion, impaired function, or necessitated revision surgery.

The outcomes observed in our cohort may be influenced by institutional practices, surgical techniques, and patient demographics unique to our center. Multicenter studies with diverse patient demographics and practices would contribute to a more comprehensive understanding of the generalizability of our findings. The continued relevance of plate osteosynthesis in PHF treatment emphasizes the need for ongoing research, technological advancements, and the integration of personalized medicine principles to optimize patient outcomes in this challenging clinical scenario.

## Conclusions

This long-term follow-up evaluation reveals no significant differences in functional outcomes or quality of life between patients treated with plate osteosynthesis and those managed non-surgically for displaced proximal humerus fractures. However, this study does not imply the irrelevance of surgical intervention, as there are still clear indications for plate osteosynthesis in humeral head fractures. It is crucial to emphasize that treatment decisions should be individualized, taking into account specific patient characteristics, clinical judgment, and patient preferences, rather than applying a one-size-fits-all approach. Shared decision-making is crucial in selecting the optimal treatment strategy for proximal humerus fractures. The observed equivalence between treatment modalities requires nuanced evaluation, considering each patient's clinical profile. Identifying the most efficacious treatment strategy should rely on a comprehensive understanding of patient-specific factors, ensuring an approach aligned with the unique circumstances of each clinical scenario.

## Data Availability

Data available upon request.
